# Breakthrough Bloodstream Infections Caused by Echinocandin-Resistant *Candida tropicalis*: An Emerging Threat to Immunocompromised Patients with Hematological Malignancies

**DOI:** 10.3390/jof6010020

**Published:** 2020-01-31

**Authors:** Maroun M. Sfeir, Cristina Jiménez-Ortigosa, Maria N. Gamaletsou, Audrey N. Schuetz, Rosemary Soave, Koen Van Besien, Catherine B. Small, David S. Perlin, Thomas J. Walsh

**Affiliations:** 1 Division of Infectious Diseases, Department of Medicine, Weill Cornell Medicine and New York Presbyterian Hospital, New York, NY 10065, USA; Sfeir@uchc.edu (M.M.S.); rsoave@med.cornell.edu (R.S.); cbs9003@med.cornell.edu (C.B.S.); 2 Department of Healthcare Policy and Research, Weill Cornell Medicine, New York, NY 10065, USA; 3 Public Health Research Institute, New Jersey Medical School/Rutgers Biomedical and Health Sciences, Newark, NJ 07103, USA; jimenecr@umdnj.edu (C.J.-O.); David.Perlin@hackensackmeridian.org (D.S.P.); 4 Department of Pathophysiology, Laikon General Hospital, Medical School, National and Kapodistrian University of Athens, 11527 Athens, Greece; magama@med.uoa.gr; 5 Department of Laboratory Medicine and Pathology, Mayo Clinic, Rochester, MN 55901, USA; Schuetz.Audrey@mayo.edu; 6 Transplantation-Oncology Infectious Diseases Program, Weill Cornell Medicine and New York Presbyterian Hospital, New York, NY 10065, USA; 7 Division of Hematology/Oncology, Weill Cornell Medicine and New York Presbyterian Hospital, New York, NY 10065, USA; kov9001@med.cornell.edu; 8 Departments of Pediatrics, and Microbiology & Immunology, Weill Cornell Medicine and New York Presbyterian Hospital, New York, NY 10065, USA

**Keywords:** *Candida tropicalis*, candidemia, echinocandin resistance, *FKS1* gene

## Abstract

Background. *Candida tropicalis* is a virulent fungal pathogen for which echinocandins are the primary therapy. Emergence of resistance to echinocandins of *C. tropicalis* carries potentially ominous therapeutic implications. Methods. We describe herein two patients with breakthrough *C. tropicalis* fungemia during echinocandin therapy, characterize their molecular mechanism of resistance, and systematically review 13 previously reported cases of echinocandin-resistant *C. tropicalis* bloodstream infections (BSIs) and other diseases. Results. Among these 15 patients with echinocandin-resistant *C. tropicalis* infections, the median age was 61 years (ages 28–84 years) and 13 (86%) were immunocompromised. Thirteen (86%) of all patients had a history of pervious or concurrent exposure to echinocandins. Isolates of *C. tropicalis* from 11 cases, including the two index cases, underwent DNA sequencing of the *FKS1* gene for mutations known to confer echinocandin resistance. The amino acid substitution Ser654Pro was shown in four cases, while other *FKS1* mutations encoded Ser80S/Pro, Phe641Leu, Phe641Ser, Ser80S/Pro substitutions. These mutational events were not associated with collateral increases in minimum inhibitory concentrations to antifungal triazoles and amphotericin B. Overall mortality in patients with echinocandin-resistant *C. tropicalis* infections was 40%. Among those six patients who died, two received monotherapy with voriconazole, one was treated with fluconazole, one remained on caspofungin, and two were switched to liposomal amphotericin B. Nine patients (60%) survived after being treated with an antifungal agent other than an echinocandin. Conclusions. Emergence of resistance to echinocandins by *C. tropicalis,* occurs during antifungal therapy, is associated with high mortality, is mediated by a diverse range of *FKS1* mutations, retains in vitro susceptibility to triazoles and amphotericin B, and constitutes an emerging threat to patients with hematological malignancies.

## 1. Introduction

Invasive candidiasis represents an important cause of morbidity and mortality among immunocompromised individuals, especially those with malignancies [1]. Echinocandins are the first-line empirical therapy of candidemia in immunocompetent and immunocompromised patients [2]. Antifungal surveillance programs demonstrate a high rate of susceptibility of *Candida* species to echinocandins in general, and specifically *Candida tropicalis* [3]. 

*Candida tropicalis* represents the third or fourth most commonly isolated non-*Candida albicans Candida* species in the clinical practice [4]. It is often found as the second most frequently isolated *Candida* species in some countries in Asia, such as Taiwan [5]. Early studies demonstrated that *C. tropicalis* was highly virulent in immunocompromised animal models and in patients with hematological malignancies [6,7]. The virulence of *Candida tropicalis* is mediated by extracellular hydrolytic enzymes, including proteinases, phospholipases, and hemolysins, which destroy the integrity of the host cell membranes [8]. Moreover, *C. tropicalis* produces biofilms, which may further amplify virulence [9]. The Transplant-Associated Infection Surveillance Network showed that *C. tropicalis* was associated with the highest all-cause mortality at 90 days (44%) compared to 26.5% for all invasive *Candida* species infections among solid organ transplant recipients in the United States [10].

Emergence of resistance to antifungal agents by pathogenic fungi poses a serious threat to the management of immunocompromised patients [11]. Little is known about echinocandin resistance in *C. tropicalis*. The aim of this study is to describe the clinical manifestations, laboratory diagnosis, molecular mechanisms of resistance, and treatment outcomes of patients with echinocandin-resistant *C. tropicalis* bloodstream infections (BSIs).

## 2. Materials and Methods

### 2.1. Literature Review 

A PubMed search was conducted to identify clinical cases with echinocandin-resistant *C. tropicalis* published through November 23, 2019. We also reviewed the reference lists of relevant studies to identify possible missed pertinent reports. Two previously unreported immunocompromised patients from our institution diagnosed with *C. tropicalis* BSIs who developed breakthrough echinocandin resistance during micafungin therapy are also described. Demographics, comorbidities, treatment, clinical outcomes, and molecular mechanisms were analyzed. 

### 2.2. Organisms 

Two isolates of *C. tropicalis* (BL37986 and BL38734) were analyzed in case 1 for *FKS* sequencing. BL37986 represents the initial isolate of *C. tropicalis* that was detected in the blood of the patient before micafungin treatment, and BL38734 was isolated from blood during micafungin therapy. The breakthrough isolate of *C. tropicalis* (isolate 2) in case 2 was obtained from blood culture on day 21 of micafungin therapy. *Candid tropicalis* within a blood culture distributes into the serum component of the sample.

### 2.3. In Vitro Antifungal Susceptibility Testing 

Antifungal susceptibility determinations against all isolates of *C. tropicalis* from both patients were performed by the Clinical and Laboratory Standards Institute (CLSI) M27-S4 methodology [12]. 

### 2.4. FKS Sequencing 

Oligonucleotide primers designed to amplify the echinocandin drug target gene *FKS1* for *C. tropicalis* isolates were based on GenBank accession number EU676168. PCR and sequencing primers are listed in Table 1. All PCR amplifications were carried out in a 50 μL reaction volume containing 50 ng of genomic DNA, 0.2 μM of each primer and 25 μL of EmeraldAmp Master MIX (Takara Bio Inc). PCR amplification conditions used for all primers were 30 cycles carried out at 95 °C for 30 s, 55 °C for 30 s and 72 °C for 3 min followed by a final extension step of 72 °C for 10 min in a BIORAD iCycler (Bio-Rad). PCR products were purified by QIAquick PCR Purification Kit (Qiagen Sciences, Inc., Germantown, MD). Automated fluorescent sequencing was performed in both 5’ and 3’ by MacroGen USA (Macrogen Corp, Rockville, MD). Sequences were assembled and edited using the SeqMan II and EditSeq software packages (Lasergene 12.0; DNAStar, Inc., Madison, WI). *C. tropicalis* ATCC 750 strain was used as the reference control. 

## 3. Results

### 3.1. Index Case 1 

The patient was an 87-year-old male with recently diagnosed left-sided upper tract urothelial carcinoma (Table 2 and Table 3). A computerized tomographic (CT)-guided biopsy of a retroperitoneal mass and left percutaneous nephrostomy showed pathology consistent with urothelial carcinoma. He underwent an elective ureteroscopy and left ureteral stent exchange in May 2015, followed by 2 cycles of neoadjuvant chemotherapy with gemcitabine and cisplatin. Following the second cycle of chemotherapy, he developed fever, left flank pain, and *C. tropicalis* (BL37986) fungemia. He was treated with intravenous fluconazole for 2 weeks. He had also acute kidney injury with a new moderate hydronephrosis, for which he underwent a right percutaneous nephrostomy. However, the patient remained febrile up to 38.2 °C at home. Two weeks later, he became critically ill, hypotensive at 65/40 mmHg, and febrile with a temperature of 39 °C. He was readmitted and received empirical therapy with intravenous vancomycin, piperacillin-tazobactam, and micafungin. 

Blood cultures detected a *C. tropicalis isolate* (BL38734) susceptible to anidulafungin (minimum inhibitory concentration (MIC) 0.06 µg/mL), micafungin (MIC 0.06 µg/mL), caspofungin (MIC 0.12 µg/mL), but resistant to fluconazole (MIC 128 µg/mL) and voriconazole (MIC 8 µg/mL). The isolate showed a low MIC of 0.5 µg/mL to posaconazole. Amphotericin B MIC was 1.0 µg/mL. He underwent emergent decompressive bilateral percutaneous nephrostomies. Urine sampled from the nephrostomy tubes identified *C. tropicalis* with the same aforementioned in vitro susceptibility profile. Micafungin was continued. Repeated blood cultures after 1 week while receiving micafungin remained positive for *C. tropicalis* (isolate BL38734) but with a new antifungal susceptibility profile demonstrating marked (≥ 16-fold) increase in MICs to all echinocandins: anidulafungin (1 µg/mL), micafungin (2 µg/mL), and caspofungin (4 µg/mL). The MICs to the remaining antifungal agents remained unchanged. Micafungin was discontinued in favor of liposomal amphotericin B at a dose of 5 mg/kg of body weight intravenous daily. Blood cultures became negative following administration of liposomal amphotericin B. The patient was successfully treated following a 14 day course. 

### 3.2. Index Case 2 

The patient was a 41-year-old female with relapsed acute myeloblastic leukemia receiving salvage chemotherapy (decitabine, then clofarabine) following an allogeneic hematopoietic stem cell transplant (Table 2 and Table 3). Patient was subsequently treated with intravenous micafungin as antifungal prophylaxis. Post-transplant, she was admitted for gastrointestinal graft versus host disease and diffuse abdominal pain associated with ascites. Her hospital course was complicated by fever, prolonged neutropenia, and *Klebsiella pneumoniae* bacteremia that was treated with intravenous meropenem. Vancomycin and micafungin were empirically added after 3 days for persistent fever following meropenem. Subsequent blood cultures collected on day 21 identified a *C. tropicalis* isolate (isolate 2) resistant to micafungin (MIC 4 µg/mL) and anidulafungin (MIC 2 µg/mL), but susceptible to fluconazole (MIC 1 µg/mL), and voriconazole (MIC 0.12 µg/mL). Amphotericin B MIC was 0.5 µg/mL) and posaconazole MIC was 0.12 µg/mL. Micafungin was discontinued and voriconazole was initiated at a dose of 6 mg/kg IV every 12 h for first 24 h then 4 mg/kg every 12 h. *Candida tropicalis* candidemia resolved on day 5 of voriconazole. However, the patient remained critically ill, developed multiorgan failure, and expired on day 27. 

### 3.3. Molecular Mechanisms of Resistance 

**Figure 1:**
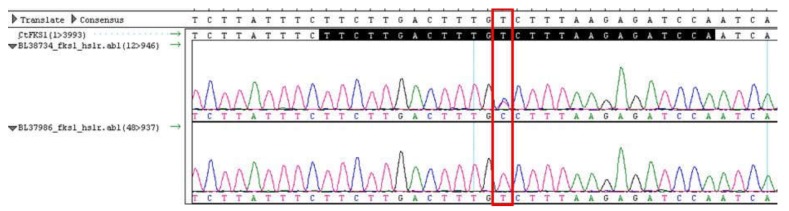
DNA sequencing chromatograms for *Candida tropicalis* BL37986 and BL38734 isolates identified in blood of Index case #1. Hot spot 1 region sequence is shown in black in the reference strain ATCC750 used as control. The red box indicates the T-to-C mutation.

**Figure 2:**
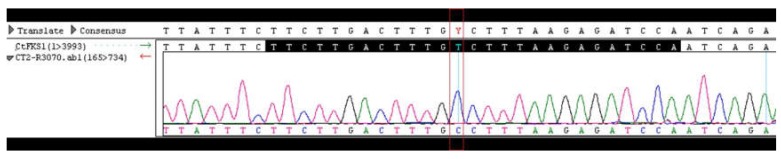
DNA sequencing chromatograms for *Candida tropicalis* isolate 2 strain isolated from blood of Index case # 2. HS1 sequence is shown in black in the reference strain ATCC750 used as control. The red box indicates the T-to-C mutation.

DNA sequencing of the two hot spot (HS) regions of the drug target gene *FKS1* known to confer echinocandin resistance was performed on three *C. tropicalis* clinical isolates: BL37986, BL38734 and isolate 2. The sequence analysis revealed a heterozygous T-to-C mutation leading to a serine-to-proline amino acid change at position 654 within the HS1 (Table 4, Figure 1) in BL38734 strain. The same mutation was found in isolate 2, but in this case in homozygosis (Table 4, Figure 2). 

### 3.4. Review of Cases

Among the 15 cases of echinocandin-resistant *Candida tropicalis* infections that developed while receiving treatment, 13 were previously reported [13,14,15,16,17,18,19,20] and two were newly described from our institution (Table 2 and Table 3). Median age was 59.5 ± 18.7 years (range 28–87) and eight (53%) patients were females. Three (20%) had acute myeloblastic leukemia, three (20%) had acute lymphoblastic leukemia, three (20%) had lymphoma, and one (7%) each had urothelial cancer, multiple sclerosis, or COPD/*Candida* lung infection. All patients except for three (20%) were immunocompromised having received chemotherapy in the last six months and three (20%) were hematopoietic stem cell transplant recipients. 

Twelve patients (80%) developed breakthrough echinocandin-resistant *C. tropicalis* candidemia with resistance defined per revised CLSI interpretive breakpoints (≥ 1 µg/mL), while receiving an echinocandin (23, 24)). Eight patients (67%) of these 12 patients had received caspofungin in the past 3 months, while four (33%) were administered micafungin. Five (42%) of those echinocandin-resistant *C. tropicalis* strains were resistant to fluconazole (MIC ≥ 8 µg/mL), four (33%) were resistant to voriconazole (MIC ≥ 1 µg/mL), and one was intermediate to voriconazole (MIC 0.25–0.5 µg/mL) based on the antifungal susceptibility testing of yeasts of the Clinical and Laboratory Standards Institute document M60 1^st^ Edition (Table 2) [21].

DNA sequencing of the *FKS1* gene for mutations known to confer echinocandin resistance was performed in 11 cases, including our two index cases (Table 2). Three cases (27%) showed the amino acid substitution Ser654Pro, one in homozygosis state (Khan AAC 2018). Three *C. tropicalis* (27%) isolates had the amino acid substitution Ser80Pro; three cases (27%) showed the amino acid substitution S645P, and one each yielded the amino acid substitution F641L and F641S, respectively.

Overall mortality was 40%. Nine patients (60%) survived after being treated with an antifungal agent other than an echinocandin. Treatment was changed to liposomal amphotericin B or voriconazole in three cases respectively, and one each to voriconazole plus caspofungin, fluconazole, voriconazole plus liposomal amphotericin B, liposomal amphotericin B plus flucytosine and caspofungin plus voriconazole. Among the six patients who died, two were treated with voriconazole, two with liposomal amphotericin B, and one with fluconazole, while one continued to receive caspofungin (Table 2). 

## 4. Discussion

This report describes the emergence of resistance of *C. tropicalis* to echinocandins in immunocompromised patients either during treatment or prophylaxis with an echinocandin. *Candida tropicalis* is a highly virulent pathogen in neutropenic hosts, where it causes increased mortality corresponding to greater degrees of tissue invasion in experimental models and immunocompromised patients with hematological malignancies [6,7,22]. The most common mechanism of resistance is a mutational event in the hot spot 1 region of the *FKS1* gene resulting in a serine-to-proline substitution at position 654. *FKS* genes encode the (1→3)-β-d-glucan synthase, which is responsible for the synthesis of the structural polymer of the fungal cell wall and is the target enzyme of the echinocandins. Hot spot mutations are known to confer elevated MICs to the echinocandins. Other antifungal agents, particularly liposomal amphotericin B, are necessary to treat these echinocandin-resistant infections caused by *C. tropicalis*.

Caspofungin was the first echinocandin approved by the Food and Drug Administration and the European Medicines Agency in 2001. Less than a decade later, Desnos-Ollivier et al. described the first case of echinocandin-resistant *C. tropicalis* with caspofungin and micafungin MICs of 2 µg/mL showing a L644W missense mutation in the hot spot 1 region of the *FKS1* gene [12]. This isolate was identified in the urine after 5 days of treatment with caspofungin at the French National Reference Center for Mycoses and Antifungals.

*Candida tropicalis* isolate BL38734 showed a point mutation involving an amino acid substitution in one of the alleles of the *FKS1* gene, while isolate 2 showed a point mutation in both alleles of the *FKS1* gene. This amino acid substitution is located in the highly conserved hot spot 1 region of the *FKS1* gene and has been associated with echinocandin resistance in *Candida* species [12]. 

In addition to our institutional cases, 13 cases of fungemia caused by *C. tropicalis* resistant to echinocandins have been reported to date [13,14,15,16,17,18,19,20]. Unlike our first patient who had a solid tumor, eight patients had hematological malignancies and five had neutropenia, which is consistent with previous studies that showed *C. tropicalis* fungemia is common among hematologic malignancies and especially in neutropenic patients [9]. 

Exposure to antifungal agents has been described as a major risk factor for drug resistance [11]. Prolonged antifungal treatment for *Candida* species infections was associated with caspofungin resistance [1,16,23,24]. Among the previously reported cases of echinocandin-resistant *C. tropicalis*, two were not exposed to echinocandin, but to voriconazole [13], and the ten remaining patients, as well as our two cases, had breakthrough resistant *C. tropicalis* BSIs after receiving an echinocandin with two of those 8 cases receiving voriconazole and caspofungin before they developed resistant *C. tropicalis* strain [14,17]. In contrast to *Candida glabrata,* where echinocandin-resistant isolates may be also be resistant to antifungal triazoles [21], resistance of *C. tropicalis* to fluconazole, other triazoles, or amphotericin B has not been a prevalent problem in immunocompromised patients. Fluconazole or amphotericin B may be used in treatment of BSIs caused by echinocandin-resistant isolates of *C. tropicalis*. However, the delay in effective therapy of echinocandin-resistant *C. tropicalis* infections in patients receiving an echinocandin may result in worsened outcome. Thus, rapid molecular identification of echinocandin-resistant C. tropicalis and other Candida spp. from the blood stream may provide life-saving information before conventional blood cultures become positive. 

To our knowledge, our index cases represent the first described echinocandin-resistant *C. tropicalis* that emerged while on micafungin (Table 2). The antecedent echinocandin-resistant *C. tropicalis* emerged while on caspofungin [13,14,16] and/or voriconazole [14,15]. Nine patients survived after changing the echinocandin to a different antifungal agent [13,14,15,16,17,18,19,20,22], in contrast to our second patient who died despite receiving voriconazole and the patient described by Garcia-Effron et al. who died after being treated with fluconazole [13]. 

The CLSI published species-specific antifungal interpretive breakpoints for resistance for echinocandins against each *Candida* species have defined an MIC of ≥1 μg/mL as non-susceptible *C. tropicalis*, an MIC of 0.5 μg/mL as intermediately susceptible, and MIC ≤ 0.25 μg/mL as susceptible *C. tropicalis* [21,23,25,26]. Unlike other *Candida* species where the MIC distributions were remarkably distinct among the three echinocandins, *C. tropicalis* MIC distributions were similar for the three echinocandins [25]. Among the 12 echinocandin-resistant isolates of *C. tropicalis*, anidulafungin appears to have the lowest in vitro MIC compared to caspofungin and micafungin (Table 2). One isolate retained in vitro susceptibility to only anidulafungin with an MIC of 0.25 μg/mL while exhibiting in vitro non-susceptibility to caspofungin and micafungin [14]. Two isolates of *C. tropicalis* were in vitro intermediately susceptible to anidulafungin and micafungin but non-susceptible to caspofungin [13,14,15,16]. Five (50%) isolates of echinocandin-resistant *C. tropicalis* were also non-susceptible to fluconazole [13,15] and only one (10%) isolate (index case #1) was also resistant to voriconazole. More investigation is required to show whether the retained in vitro anidulafungin activity and/or azoles susceptibility are correlated with different clinical outcomes and whether mutations are heterozygous or homozygous result in all mutant enzymes or 50% wild type and mutant. 

In conclusion, *C. tropicalis* is a highly virulent *Candida* species that is usually susceptible to echinocandins. However, emergence of resistance may develop following exposure to echinocandin treatment. The resistance is most commonly caused by a serine-to-proline substitution in the known hot spot 1 region of *FKS1*. Liposomal amphotericin B is an effective empirical alternative in patients with hematological malignancies pending susceptibility testing [24]. Finally, continuous surveillance in institutions for antifungal resistance of *C. tropicalis* and other *Candida* species is highly recommended, as emerging trends in resistance may serve as a basis for changing patterns of practice in the use of echinocandins.

## Figures and Tables

**Table 1:** Oligonucleotides used for *FKS1* PCR and sequencing.

Organism	Hot Spot 1 Forward Primer (5’-3’)	Hot Spot 1 Reverse Primer (5’–3’) *	Hot Spot 2 Forward Primer (5’–3’) *	Hot Spot 2 Forward Primer (5’–3’)
*C. tropicalis*	AATGGGCTGGTGCTCAACAT	CCTTCAATTTCAGATGGAACTTGATG	AAGATTGGTGCTGGTATGGG	TAATGGTGCTTGCCAATGAG

* primers used for sequencing.

**Table 2:** Characteristics of the cases of echinocandin-resistant *Candida tropicalis* infections.

Published Case, Year of Publication	Age	Sex	Underlying Condition	Source	Antifungal Treatment in the Previous 3 Months	ANF MIC	CAS MIC	MCF MIC	FLU MIC	ITR MIC	VOR MIC	POS MIC	AMP MIC	5FC MIC	FKS1 Hot Spot Sequence Analysis	Antifungal Treatment	Outcome
Grosset M. et al., *Med Mycol Case Rep.* 2016	37	M	Tuberculosis	blood	caspofungin then voriconazole	0.5	4	0.5	0.5	NA	0.06	0.06	0.06	0.124	FKS1-S645P	liposomal amphotericin B and flucytosine then voriconazole	survived
Jensen R. et al., *Antimicrob Agents Chemother*. 2013	51	F	ALL	blood	voriconazole then caspofungin	0.25	>32	1	0.5	≤0.03	≤0.03	≤0.03	0.5	NA	FKS1-S80S/P	amphotericin B	survived
Garcia-Effron G. et al., *Antimicrob Agents Chemother*. 2008	84	M	ALL	blood	caspofungin	2	4	2	8	0.5	1	0.25	NA	NA	FKS1- S645P	voriconazole	survived
Garcia-Effron G. et al., *Antimicrob Agents Chemother*. 2008	59	M	Large-cell lymphoma-allo-HSCT	blood	caspofungin	1	4	2	32	1	2	1	NA	NA	FKS1- F641L	liposomal amphotericin B	survived
Garcia-Effron G. et al., *Antimicrob Agents Chemother*. 2008	45	M	Hodgkin lymphoma, renal cell carcinoma, esophageal cancer	blood	caspofungin	0.5	1	0.5	0.5	0.125	0.03	0.06	NA	NA	FKS1- S645P	fluconazole	died
Garcia-Effron G. et al., *Antimicrob Agents Chemother*. 2010	28	F	AML	blood	caspofungin	1	4	1	1	NA	NA	NA	NA	NA	FKS1-F641S	fluconazole	survived
Pfeiffer CD et al., *J Clin Microbiol*. 2010	65	F	Bilateral lung transplant recipient	blood	micafungin	4, 2	8, 4	2,2	NA	NA	NA	NA	NA	NA	FKS1- S80S/P	amphotericin B lipid complex	died
Pfeiffer CD et al., *J Clin Microbiol.* 2010	45	F	Ventral hernia	blood, pleural fluid	micafungin	0.12	0.25	0.06	NA	NA	NA	NA	NA	NA	None	amphotericin B lipid complex	died
Kofteridids D. et al., *J Antimicrob Chemother*.2010	60	M	Large B cell lymphoma-HSCT	blood	voriconazole	NA	8	NA	32	NA	1	2	0.5	NA	NA	liposomal amphotericin + voriconazole	survived
Kofteridids D. et al., *J Antimicrob Chemother.* 2010	66	F	AML- HSCT	blood	voriconazole	NA	16	NA	1	NA	0.2	0.06	0.5	NA	NA	voriconazole	survived
Kofteridids D. et al., *J Antimicrob Chemother.* 2010	84	M	ALL, prostate cancer	blood	caspofungin	NA	8	NA	8	NA	0.25	0.2	0.5	NA	NA	caspofungin + voriconazole	survived
Khan Z. et al., *Antimicrob Agents Chemother*. 2018	34	F	Multiple sclerosis on natalizumab	endotracheal secretions (two *C. tropicalis* isolates)	caspofungin	1; 1 *	16; 32 *	0.5; 075 *	1; 0.5*	0.031; 0.063 *	0.063; 0.125 *	0.063; 0.016 *	0.25; 0.25*	NA; NA*	FKS1-S654P (homozygous)	caspofungin	died
Xiao M. et al., Inf *Drug Resis.* 2018	69	F	Asthma, pulmonary infection, coronary artery disease, multiple organ dysfunction	chest drainage	micafungin x18 days	2	4	2	2	0.25	0.125	0.25	0.5	≤0.06	FKS1-S80P	voriconazole	died
Index case #1	87	M	Urothelial carcinoma	blood	micafungin	1	4	2	128	NA	8	0.5	1	0.12	FKS1-S654P	liposomal amphotericin B	survived
Index case #2.	41	F	AML	blood	micafungin	2	16	4	1	NA	0.12	0.12	0.5	0.12	FKS1-S654P	voriconazole	died

Abbreviations: NA: not available; ALL: acute lymphoblastic leukemia; AML: acute myeloblastic leukemia; allo-HSCT: allogeneic hematopoietic stem cell transplant; ANF: anidulafungin; CAS: caspofungin; MCF: micafungin; FLU: fluconazole; ITR: itraconazole; VOR: voriconazole; POS: posaconazole; AMP: amphotericin B; 5FC: flucytosine; MIC: minimum inhibitory concentration in µg/mL; M: male; F: female; TPN, total parenteral nutrition. * Correspond to MIC of second *C. tropicalis* isolate.

**Table 3:** Demographics and clinical characteristics of patients with echinocandin-resistant *Candida tropicalis* bloodstream infections.

Characteristic	*n* = 15 (%)
Age, years, median ± SD	59.5 ± 18.7
Gender	
Male	7 (47)
Female	8 (53)
Comorbidities	
ALL *	3 (20)
AML	2 (13)
Lymphoma *	3 (20)
Urothelial cancer	1 (7)
Multiple sclerosis	1 (7)
COPD/*Candida* lung infection	1 (7)
Prior exposure to echinocandins	13 (87)
Echinocandin resistance occurrence	
Breakthrough resistance while on echinocandin	12 (80)
*De novo* resistance (nil exposure to echinocandins)	2 (13)
Outcome	
Recovery	9 (60)
Death	6 (40)

Abbreviations: ALL: acute lymphoblastic leukemia; AML: acute myeloblastic leukemia. * One patient had prostate cancer in addition to ALL and another patient had renal cell carcinoma and esophageal malignancy concomitantly with lymphoma.

**Table 4:** *FKS1* Hot Spot1 sequencing of the *Candida tropicalis* isolates.

*Candida tropicalis* Isolate	DNA Sequence	Protein
ATCC 750 Ref strain	TTCTTGACTTTGTCTTTAAGAGATCCA	FLTLSLRDP
BL37986	TTCTTGACTTTGTCTTTAAGAGATCCA	FLTLSLRDP
BL38734	TTCTTGACTTTGCCTTTAAGAGATCCAA	FLTLS/PLRDP
Isolate 2	TTCTTGACTTTGCCTTTAAGAGATCCAA	FLTLPLRDP

The red letters designate the mutations at the nucleotide and amino acid level.
